# P-1136. Enhancing Pediatric Care: Antimicrobial Stewardship Through 24/7 Pharmacist-Driven Rapid Diagnostics for Gram-Negative Bacteremia

**DOI:** 10.1093/ofid/ofae631.1323

**Published:** 2025-01-29

**Authors:** Anthony Huynh, Paul Gavaza, Noreen Chan Tompkins

**Affiliations:** Loma Linda University School of Pharmacy, Loma Linda, California; Loma Linda University School of Pharmacy, Loma Linda, California; Loma Linda University Children's Hospital & School of Pharmacy, Loma Linda, California

## Abstract

**Background:**

Rapid diagnostic testing (RDT) in adult bacteremic patients (pts) provides quick organism identification, including resistance genes and can improve time to optimal antibiotic therapy (OAT). Pharmacist-driven RDT (Rx-RDT) interventions [24 hours/day and 7 days/week (24/7)] for pediatric gram-negative bacteremia GNB is limited. Objective was to evaluate outcomes before and after implementation a 24/7 Rx-RDT interventions (INT) for treatment of pediatric GNB.

**Figure:**
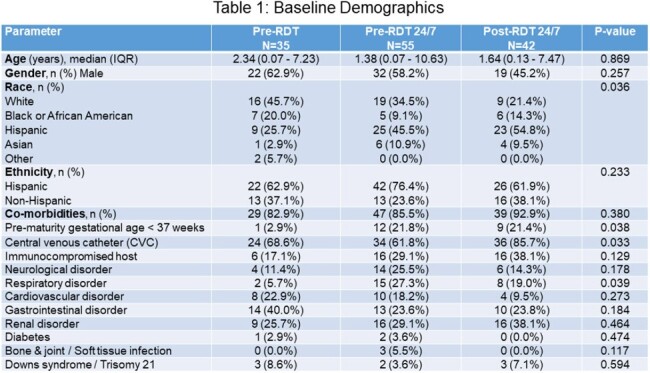

**Methods:**

Retrospective cohort study analyzed pediatric pts (age < 18 years) with GNB secondary to *Escherichia coli, Klebsiella pneumoniae* or *Klebsiella oxytoca* admitted to the Children’s Hospital comparing outcomes before and after implementation of 24/7 Rx-RDT INT. Pre-RDT group included pts admitted from 1/1/14-12/31/14; Pre-RDT 24/7 group embodied weekday antimicrobial stewardship (AMS) duties from 1/1/19-7/5/21; and Post-RDT 24/7 from 7/6/21-8/31/22. Polymicrobial bacteremia or concomitant fungemia were excluded.

**Results:**

A total of 132 pts were included in 3 groups: Pre-RDT (n=35); Pre-RDT 24/7 (n=55); Post-RDT 24/7 (n=42). Baseline demographics were mainly similar with Hispanic predominance (Table 1). In Pre-RDT, Pre-RDT 24/7, and Post-RDT 24/7, respectively, *E. coli* was most common (65.7% vs 60% vs 54.8%; p=0.666) with CTX-M gene detection (associated with the extended spectrum beta-lactamase (ESBL) phenotype) in latter 2 groups (9.1% vs 26.1%; p=0.013) followed by *K. pneumoniae* (28.6% vs 25.5% vs 33.3%; p=0.666) with ESBL 21.4% in latter groups; p >0.05). Pts were on OAT at the time of gram-stain (GS) results in 85.7% vs 74.5% vs 59.5%; p=0.034. For pts who were not on OAT at GS results, time to OAT from blood culture collection was lowest in the Post-RDT 24/7 group (85.6 vs 26.2 vs 15.7 hours; p=0.156); time to OAT was faster from time of RDT results Post-24/7 RDT (7.9 vs 2.4 hours; p=0.028). Length of stay (19.5 vs 12.9 vs 13.3 days; p=0.601) and 30-day mortality were similar (11.4% vs 14.5% vs 9.5%; p=0.746); 30-day mortality occurred with Hispanics in all groups (18.2% vs 16.7% vs 14.4%). Night/Weekend pharmacists performed 44% of the Post-RDT 24/7 INT.

**Conclusion:**

Implementation of Rx-RDT interventions 24/7 reduced time to OAT by 30% for pediatric GNB. Night/Weekend pharmacists were integral to our AMS program.

**Disclosures:**

**All Authors**: No reported disclosures

